# Aspermy, Sperm Quality and Radiation in Chernobyl Birds

**DOI:** 10.1371/journal.pone.0100296

**Published:** 2014-06-25

**Authors:** Anders Pape Møller, Andrea Bonisoli-Alquati, Timothy A. Mousseau, Geir Rudolfsen

**Affiliations:** 1 Laboratoire d’Ecologie, Systématique et Evolution, CNRS Unité Mixte de Recherche 8079, Université Paris-Sud, Bâtiment 362, Orsay Cedex, France; 2 University of South Carolina, Department of Biological Sciences, Columbia, South Carolina, United States of America; 3 Department of Arctic and Marine Biology, University of Tromsø, Tromsø, Norway; National Cancer Institute, United States of America

## Abstract

**Background:**

Following the Chernobyl nuclear power plant accident, large amounts of radionuclides were emitted and spread in the environment. Animals living in such contaminated areas are predicted to suffer fitness costs including reductions in the quality and quantity of gametes.

**Methodology/Principal Findings:**

We studied whether aspermy and sperm quality were affected by radioactive contamination by examining ejaculates from wild caught birds breeding in areas varying in background radiation level by more than three orders of magnitude around Chernobyl, Ukraine. The frequency of males with aspermy increased logarithmically with radiation level. While 18.4% of males from contaminated areas had no sperm that was only the case for 3.0% of males from uncontaminated control areas. Furthermore, there were negative relationships between sperm quality as reflected by reduced sperm velocity and motility, respectively, and radiation.

**Conclusions/Significance:**

Our results suggest that radioactive contamination around Chernobyl affects sperm production and quality. We are the first to report an interspecific difference in sperm quality in relation to radioactive contamination.

## Introduction

The primary function of sperm is to ensure fertility and enhance paternity, and consequently strong selection acts on both qualitative and quantitative sperm traits [1]. Recent studies of sperm behavior have revealed a close correlation between qualitative sperm traits and fertilization success in a wide range of taxa [2]–[9]. Furthermore, it is now clear that the production of high-quality ejaculates may represent significant costs of reproduction for males [10]–[13], and that sperm quality and production are related to several factors [14]–[17], including external factors [18]–[25].

Following the Chernobyl nuclear power plant accident in 1986, large quantities of radionuclides were emitted and spread in the environment. Previous studies have reported reproductive failure, such as increased infertility, early impotence, and reduced total number of sperm including reduced number of motile sperm among clean-up workers and men living in contaminated regions [18], [21], [23], [26]–[29]. Furthermore, high doses of x-rays lead to permanent sterility in humans [30], [31], while low doses do not [32]. However, persistence of ^137^Cs contamination raises questions regarding long-term effects on reproduction and fertility. Possible mechanisms underlying the effect of radiation on sperm parameters are direct damage to DNA integrity [23] or indirectly through excess reactive oxygen species (ROS) [33], [34]. Ionizing radiation can cause oxidative stress by increasing the production of ROS, which renders antioxidants incapable of defense against increasing amounts of free radicals [35], [36].

Increased rates of DNA damage and mutation rates have been reported from Chernobyl [23], [37]–[45]. Admittedly, the literature reports heterogeneous results on DNA damage and mutation rate in organisms living in areas around Chernobyl with low levels of contamination [46]–[49]. However, the susceptibility of sperm to DNA damage stems partly from the down-regulation of DNA repair systems during late spermatogenesis [24]. As a consequence, ejaculated sperm may exhibit genetic damage to both nuclear and mitochondrial genomes [24], [25].

High seminal ROS levels lead to oxidative stress that in turn can reduce sperm quality and increase sperm DNA fragmentation rates [50]–[52]. The susceptibility of sperm cells to oxidative stress is partly due to their limited antioxidant machinery and high metabolic activity [33], [53]–[55]. In addition, oxidative stress stimulates a lipid peroxidation cascade in the plasma membrane that in particular influences sperm motility [56]–[58].

Previous studies of barn swallows *Hirundo rustica* from a variety of locations in Ukraine have revealed a positive correlation between background radiation level and the frequency of sperm with abnormal morphology and impaired sperm motility [44], [59]. There is also empirical evidence for birds breeding in heavily contaminated areas around Chernobyl having increased mutation rates, higher oxidative stress and higher incidence of morphological aberrations [42]–[44], [59]–[61]. Hence, results from field studies on free-living birds seem to be consistent with the theoretical background on the effects of radiation on sperm production and performance.

The potential fertility of individuals can be assessed from measurements of semen quantity and quality, and such analyses provide an umbrella endpoint for a wide spectrum of processes reflecting the functional state of spermatogenesis. In addition, gametogenesis is known to be sensitive to radiation [62], and damage in the germ line can impair fitness. Consequently, both sperm production and sperm motility act as an interesting endpoint when examining the effect of radiation on wild populations. We recorded aspermy (absence of sperm in the seminal fluids) and sperm behavior with a computer assisted sperm analyzer (CASA) in a large sample of birds under field conditions at Chernobyl. The biological effects of radiation may vary among taxonomic groups [63], and that is also the case for sperm parameters [31], [64]. In addition, bird species are found to vary in sperm motility [65], [66]. Thus, we predicted that both presence of sperm production (lack of aspermy) and sperm behavior would be negatively associated with background radiation.

## Methods

### Ethics Statement

The research complied with requirements for research on birds in Ukraine, and permission was given by the administration of the Chernobyl Exclusion Zone. All sampling was approved in an ethical review by the University of South Carolina Institutional Animal Care and Use Committee. All birds were handled briefly, and none died or showed signs of suffering during the short examination. All individuals flew upon release. The field studies did not involve endangered or protected species.

### Study Sites and Bird Capture

**Figure 1:**
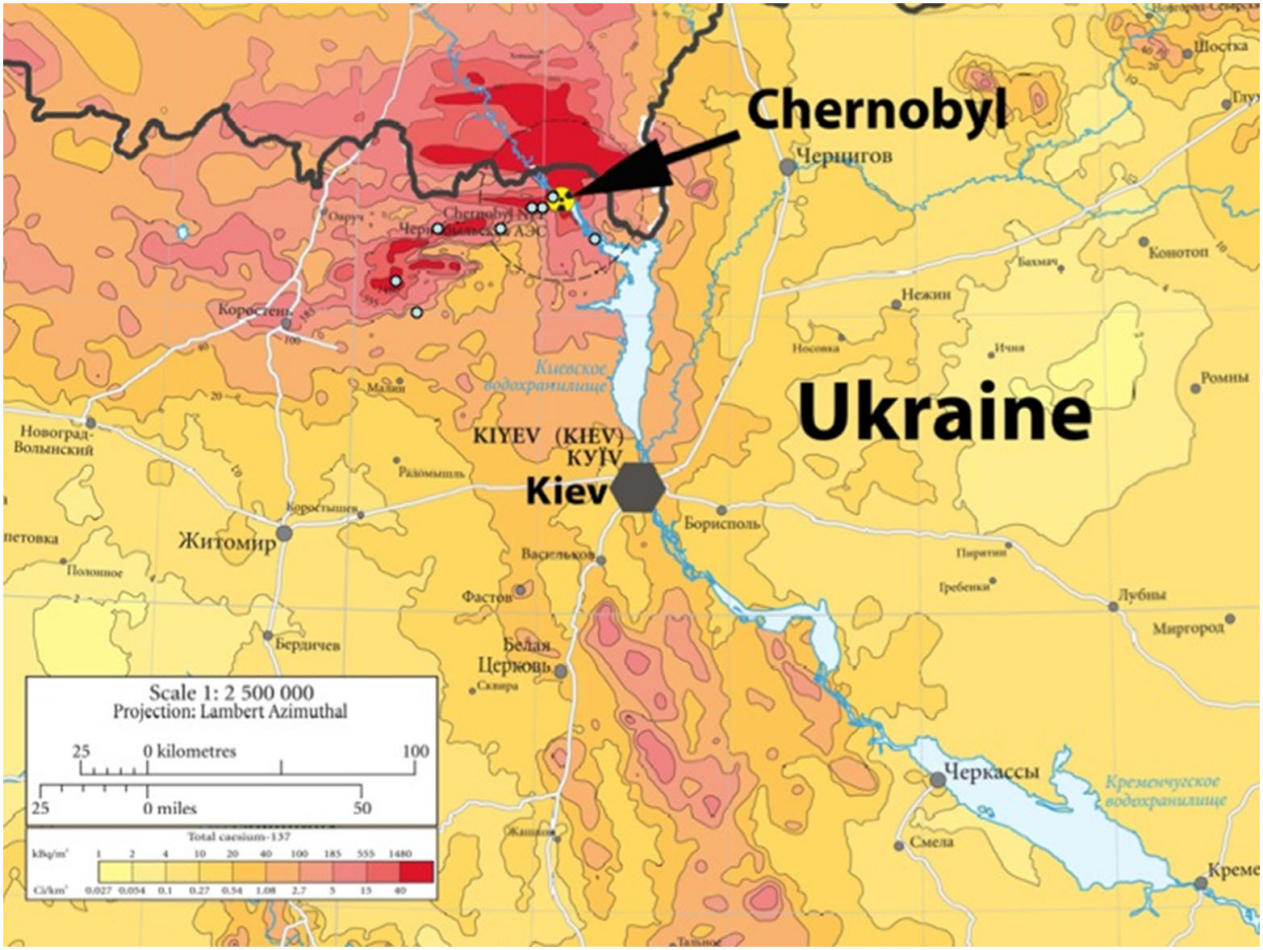
Map of background radiation (µSv/h) in the Chernobyl region and location of the study sites. Adapted from European Union [77].

We captured a total of 566 males out of a total of 1076 birds using 35 12 m mist nets placed in woodland habitat and farms in 8 different sites around Chernobyl, Ukraine during 25 May-5 June 2010 and 30 May-9 June 2011. Each site was used for capture of birds for two consecutive days covering both early morning and late evening, except for barns where a single capture session was performed for barn swallows. Sites were chosen to cover a wide range of deposition of radionuclides while still being located in the vicinity of the most contaminated areas in the exclusion zone of Chernobyl (Fig. 1). A total of 566 passerine males were examined for sperm. We measured background radiation levels at the exact capture sites of each individual by using a hand-held dosimeter (Inspector, SE International, Summertown, Tennessee, USA), which is sensitive to low levels of alpha, beta, gamma, and x-rays. Although there are significant differences in background radiation among sites (*F* = 1253, d.f. = 7, 279, *P*<0.001), there is considerable variation within each site (Table S2). Thus, we used background radiation at the exact trapping position of each individual bird as a predictor of external exposure, and radiation levels varied from 0.02 to 137.9 µSv/h. Two sites were assigned as control sites because all trapping positions had ≤0.01 µSv/h as background radiation. Previous studies have shown a strong positive relationship between internal and external dose of birds in this study area accounting for more than two thirds of the variance [67].

### Morphological Characters, Sex and Age

We sexed and aged birds according to standard criteria [68], although the lack of reliable aging criteria prevented us from aging all species. Body mass was recorded with a Pesola spring balance to the nearest 0.1 g while keel length (as a measure of structural body size) was recorded with a digital caliper to the nearest 0.01 mm.

### Ejaculate Samples

Subsequent to capture of males APM gently massaged the cloacal protuberance of 566 passerine males of which 490 provided an ejaculate and then released the birds following measurements and blood sampling [69]. Sperm were collected in micro-capillaries for easy handling. Ejaculates can be collected from free-living wild birds by gentle massage resulting in contraction of the muscles surrounding the cloaca, and a small ejaculate emerging can subsequently be collected in a micro-capillary tube. There is a strict temporal coincidence of the contraction of muscles and the appearance of the ejaculate. In the absence of semen, a second and a third attempt were made with males still showing no sign of sperm being classified as cases of aspermy. This was justified because in the cases where we found no sperm in an ejaculate, there was still a clear contraction of muscles. When APM made a second or a third attempt, there was still an obvious contraction of the cloacal muscles and any substance collected from the cloaca in most cases still did not contain sperm. When such collection of ejaculates was made in uncontaminated areas near Chernobyl or in Denmark or Spain, ejaculates were almost invariably collected in the first attempt. The number of individuals with and without sperm are listed in Table S1.

When there was sperm in the sample, the ejaculate was immediately diluted in 20–100 µl, depending on the size of the ejaculate, preheated Dulbecco’s Modified Eagle Medium (Invitrogen, Carlsbad, CA). Within 1 min after ejaculation, about 4–5 µl of the diluted sperm were pipetted onto a preheated standard microscopic count slide (20 µm depth two-chamber, Leja, Nieuw-Vennep, The Netherlands) mounted on a MiniTherm slide warmer (Hamilton Thorne Inc., Beverly, MA) kept at a temperature of 39°C, which is close to the mean temperature of 41.8°C (SE = 0.06). Sperm behavior was then recorded for multiple independent frames for each slide to increase the number of sperm measured for each male using a Sony CCD black-and-white video camera (XC-ST50CE PAL, Sony, Tokyo, Japan), mounted on an external negative phase-contrast microscope (Olympus CH30, Olympus, Tokyo, Japan) with a 10x objective. The recordings were later analyzed with computer-assisted sperm analysis (CASA, HTM-CEROS sperm tracker, CEROS v.12, Hamilton Thorne Research, Beverly, MA, USA), which allows the recording of several standard motility measurements, including (1) VSL (straight line velocity), (2) VCL (track velocity), (3) VAP (curvilinear velocity), (4) ALH (amplitude of lateral head displacement), (5) BCF (beat cross frequency), (6) LIN (linearity, ratio of VSL/VCL) and (7) STR (Straightness = ratio of VSL/VAP). These measurements were used to estimate the percentage of static sperm, and sperm with slow, medium and high velocity. In total this provides 13 descriptors of sperm behavior.

### Statistics

Odds-ratio statistics with 95% confidence intervals were obtained from JMP [70] and were used to compare age and frequency of aspermy in contaminated areas versus controls. We predicted the presence or absence of sperm using logistic regression models with species, radiation level, age, body mass and keel length as predictors. When both body mass and keel length are entered into the same model, the body mass effect can be interpreted as a measure of body condition. We compared absence of sperm in different samples using contingency analyses. We used Welch’s ANOVA of unequal variances and Levene’s test [70] to assess differences in mean and variance in radiation between males with and without sperm.

Prior to our analysis of sperm behavior, we excluded species that were only trapped at a single site (see Table S2). Furthermore, all sperm traits were quantified as the mean value for each male across several video frames, weighted by the total number of cells for the actual frame. As different sperm traits reported from CASA are strongly correlated, we quantified sperm traits by using a principal component analysis of the 13 ejaculate parameters described above, relying on the varimax approach on the correlation matrix with Kaiser normalization [70]. This reduced the correlated variables to three statistically independent axes that reflected different aspects of overall sperm traits (Table S3). Repeatabilities of the three principal components and the associated statistics are reported in Table S4.

Relationships between the three principal components of sperm and levels of background radiation were analyzed in mixed models with log-transformed external radiation as a fixed factor and species as a random factor. As sperm traits among different bird species are known to vary [65], [66], we assumed that their response to radiation could also vary. Consequently we followed standard guidelines [69] and ran both a model where each species could vary both in terms of intercept and slope, and a simpler model where species could only vary in their intercept. Model fitting and estimates were obtained with JMP [70] using restricted maximum likelihood estimates (REML). The significance of including the varying slope parameters was tested using log-likelihood ratio statistics, with the use of AICc (Akaike’s Information Criterion) [70]. Finally, the model fit was verified using visual examination of normal probability plots and residual plots.

## Results

### Aspermy

**Figure 2:**
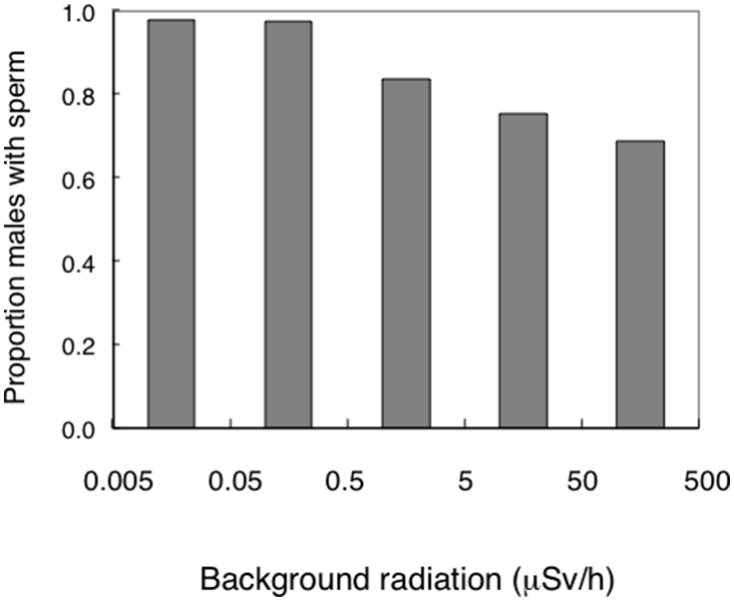
Box plots of background radiation level (µSv/h) for birds without and with sperm. Values are medians, quartiles, 5- and 95-percentiles and extreme values.

**Figure 3:**
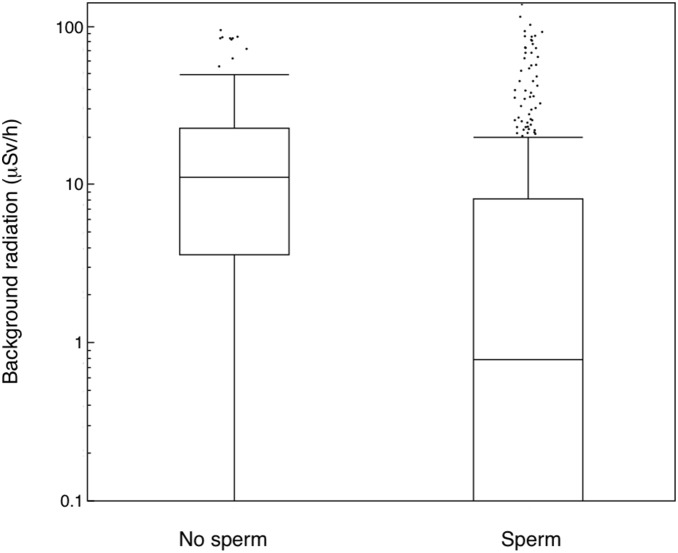
Proportion of birds with sperm in relation to background radiation level (µSv/h).

A total of 76 out of 566 male birds that were examined for sperm had no sperm, corresponding to 14.0%. In control areas 5 out of 164 (3.0%) individuals suffered from aspermy, while 74 out 402 (18.4%) individuals did so in contaminated areas. Odds ratio calculation suggests that it is 8.9 (3.0–35.7, 95% CI, *P*<0.001) times more likely to find aspermy in males caught in contaminated areas compared to uncontaminated control areas (Fig. 2). Indeed, both mean and variance in exposure differed between males with aspermy and sperm (Welch ANOVA for unequal variances: *F* = 109.64, d.f. = 1, 143.29, *P*<0.0001; Levene’s test: *F* = 104.70, d.f. = 1, 564, *P*<0.0001). Young birds were more common in contaminated than in control areas (61.3% of 256 individuals in contaminated areas, 49.2% of 128 individuals in control areas, odds ratio = 2.2 (1.4–3.4, 95% CI, *P*<0.001)). However, the main predictor of absence of sperm was background radiation level, showing a decrease in the proportion of males having sperm with increasing radiation level (Table 1; Fig. 3). The effects of species and age were not significant (χ^2^ = 37.05, d.f. = 31, *P* = 0.21, χ^2^ = 0.0003, d.f. = 1, *P* = 0.99, respectively). A full model that also included keel length and body mass to account for effects of body condition in addition to radiation showed an overall highly significant model that accounted for 20% of the variance (Table 1 model 2). The most important predictor was radiation implying that males without sperm were more common at higher levels of radiation. In addition, there was a weak effect of body mass, suggesting that males in poor condition more often had no sperm (Table 1 model 2).

**Table 1:** Absence of sperm in relation to level of background radiation (model 1) and level of background radiation, keel length and body mass (model 2).

Variable	χ^2^	d.f.	*P*	Slope (SE)
**Model 1:**				
Radiation	39.90	1	<0.0001	−0.835 (0.132)
Year	7.14	1	0.0075	0.359 (0.137)
**Model 2:**				
Radiation	55.28	1	<0.0001	−0.872 (0.138)
Keel length	8.56	1	0.0034	8.590 (2.970)
Body mass	3.73	1	0.053	−2.281 (1.188)
Year	3.20	1	0.074	−0.259 (0.146)

The models had the statistics χ^2^ = 68.74, d.f. = 2, *r* ^2^ = 0.15, *P*<0.0001 and χ^2^ = 81.44, d.f. = 4, *r* ^2^ = 0.18, *P*<0.0001.

### Sperm Behavior and Radiation

We obtained recordings of live sperm from 399 males belonging to 24 different species and in at least two different sites for each species, and these two sites were always an uncontaminated and a contaminated site (Table S2). Species with captures at only a single site are listed in Table S2. A principal component analysis of the CASA measurements of sperm behavior reduced the number of variables to three uncorrelated principal components accounting for 75.9% of the variance (Table S3). PC1 was highly positively correlated with all sperm velocity and motility variables. Furthermore, this principal component had strong loadings for sperm that were swimming straight and linearly (Table S2). PC2 reflected ejaculates having linear movements, and were motile, progressive and with a high fraction of rapid, medium and slow sperm thus reflecting ejaculates with motile sperm, but reduced velocity (Table S3). PC3 represented sperm with a high beat cross frequency and a high degree of straight swimming and many sperm with medium, slow and static sperm (Table S3). Thus, PC3 represented ejaculates with strongly reduced forward motion.

Analysis of the first principal component explaining 39.6% of the variance and that strongly correlated with sperm velocity and progressive sperm showing that there were significant species-specific effects (Table 2). There was no significant main effect of radiation (Table 2), while there was a significant interaction between species and radiation level and a significant year effect (Table 2). This interaction effect implies that sperm behavior was not consistently negatively correlated with radiation across all species; some species showed no significant radiation effect while others showed strong radiation effects.

**Table 2:** Generalized linear mixed models of the relationship between three principal components of sperm behavior and radiation, species×radiation and year.

Variable	d.f.	*F*	*P*	Estimate	SE
**PC1**					
Radiation	1,221.1	1.15	0.28		
Species×Radiation	23,284.7	2.27	0.0010		
Year	1,368.4	321.49	<0.0001		
**PC2**					
Radiation	1,205.4	5.93	0.016	0.241	0.099
Species×Radiation	23,283.0	1.61	0.041		
Year	1,367.3	523.97	<0.0001		
**PC3**					
Radiation	1,237.4	0.18	0.67		
Species×Radiation	23,235.6	2.08	0.0034		
Year	1,370.9	0.11	0.74		

The random species effects were as follows: PC1: variance component 0.585, 95% CI = 0.1081, 1.0612, accounting for 21% of the variance. PC2: variance component 0.360, 95% CI = 0.0694, 0.6501, accounting for 24% of the variance. PC3: variance component 0.095, 95% CI = −0.0631, 0.2533, accounting for 7% of the variance.

Analysis of the second principal component that accounted for 24.2% of the variance and that correlated with sperm having medium velocity, showed a significant species-specific slope in relation to radiation level (Table 2). We found a significant effect for PC2 in relation to radiation (Table 2). In addition, there was a significant interaction between radiation and species (Table 2) and an effect of year (Table 2). This interaction effect implies that sperm behavior was not overall negatively correlated with radiation in all, but only in some species.

The third principal component, explaining 12.0% of the variance in sperm traits, representing ejaculates with strongly reduced forward motion, showed a species-specific response to radiation (Table 2). However, there was no significant main effect of radiation (Table 2), nor a significant year effect (Table 2). The interaction between species and background radiation was statistically significant (Table 2). This interaction effect implies that sperm behavior was not overall negatively correlated with radiation in all but only in some species.

## Discussion

The main findings of our study were, firstly, that aspermy is more common in birds living in contaminated areas near Chernobyl than in uncontaminated control areas. Second, among males that produce sperm there was a general decrease in sperm velocity and motility in relation to radioactive contamination around Chernobyl.

This study is by definition correlational. A similar experimental study under field conditions would not be possible for ethical and logistic reasons. However, this is by far the largest study of sperm in birds under field or laboratory conditions, and we are unaware of even a single other study investigating the effects of ionizing radiation on sperm in birds. Our study was conducted on free-living birds that live under more challenging natural conditions such as common food and nutrient restriction and the adverse effects of predation, parasitism and competition, as compared to the often benign and sheltered conditions experienced by animals in the lab.

There were 79 males that did not have any sperm either due to sperm depletion or aspermy. Levels of background radiation predicted aspermy for birds caught in the surroundings of Chernobyl, with more males without sperm being found at high levels of background radiation. We found no significant heterogeneity among species, suggesting that these severe effects of radiation on aspermy had a common ecological background. This is surprising given that the sex ratio is more male biased at higher levels of radiation [71]. Hence, males in contaminated areas should on average compete more strongly for mates, but have fewer mating opportunities and thus less frequently have been depleted of sperm. This is opposite to the observed effect.

The high level of aspermy of up to 40% at the highest radiation levels is by any measure very large when compared to natural levels. There are only few comparable data for other organisms including humans. Lifjeld et al. [72] showed that 4% of willow warblers *Phylloscopus trochilus* and 2% of bluethroats *Luscinia svecica* had no sperm in the seminal glomera during the breeding season, which is similar to what we found in the uncontaminated control areas. In addition, the total number of sperm and the number of motile sperm has been found to be lower in Chernobyl victims than in controls [23].

Sperm behavior in birds living in the vicinity of Chernobyl appears to be affected by radiation, and our results are consistent with previous studies of sperm traits in birds at Chernobyl [59]. In addition, survival rate of adult birds is reduced in Chernobyl compared to nearby control areas suggesting that only males of high quality survive [71]. Thus, the estimated effect of radiation on sperm quality and quantity should be considered as conservative. Despite this likelihood, the sperm velocity was reduced among 24 species that were sampled in at least two different locations. This result is unlikely to be due to differences in reproductive timing as there is no indication of differences in laying date between Chernobyl and Ukrainian control areas [72].

We found large year effects for PC1 and PC2, but not for PC3. These year effects may arise for a number of reasons including differences in population density, food abundance or climate among years. We have no possibility for discriminating among these explanations since none of these factors appeared to vary consistently among years.

The species-specific effect of radiation on all three components of sperm behavior seems to follow a pattern that bird species having relatively high frequency of poorly performing sperm in areas with low background radiation are less sensitive to radiation than species that have low frequency of such sperm in areas with low background radiation. We are the first to report such an effect on sperm behavior among individuals from wild populations, although the result is in line with other studies showing that there may be species-specific differences in biological effects of radiation [63]. However, the standard errors indicate large intraspecific variation (Table 2) and thus care should be taken when interpreting the slope. However, the present result is consistent with previous findings for large samples of barn swallows from Chernobyl [59].

Selection should act on traits important for fitness, and consequently males should allocate resources and trigger mechanisms underlying sperm function aimed at avoiding oxidative stress and preventing oxidative damage to sperm structures. Barn swallows in Chernobyl do not seem to protect their sperm from oxidative stress and they have elevated levels of ROS [60]. The observed effect of radiation on sperm reported in this study is likely attributable to production errors during spermatogenesis, may result from increased perioxidation of the sperm membrane after formation [58], or may be due to acrosome damage [73]. The most likely mechanism would be increased exposure to radiation due to the Chernobyl accident.

The negative effect of high doses of radiation on intra- and intercellular ROS levels is well known [74], while the low dose effect of radiation at the individual level is less understood [75]. The observed negative effect of radiation on reproductive traits in birds at the individual levels may help explain the observed decline in wild bird populations in the most contaminated areas in Chernobyl [76]. In addition, absence of sperm from males inhabiting the most radioactively contaminated areas will prevent the spread of mutations from such areas. Mutation rates around Chernobyl are elevated by up to more than a factor twenty [42]. The underlying mechanisms responsible for the inter-specific response of sperm to radiation may help to understand mechanisms that influence sperm production errors and maintain sperm integrity under extreme environmental perturbations. Finally, knowledge about the species-specific response to radiation may be important for management following nuclear accidents. In summary, the effect of radioactive contamination on sperm shows how external environmental perturbations could impair traits that are crucial for reproductive success and hence fitness.

## Supporting Information

Table S1 **Bird species and number of individuals with aspermy and with sperm around Chernobyl.**
(DOC)

Table S2 **Bird species and number of individuals sampled for sperm around Chernobyl.**
(DOC)

Table S3 **Principal component analysis of 13 sperm parameters recorded with CASA.** Rotation method: Varimax with Kaiser Normalization. Loadings above 0.4 are shown in bold.
(DOC)

Table S4 **Repeatability (** ***R*** **) of the three principal components of sperm behavior.**
(DOC)
