# Supplementary material for: Aspermy, Sperm Quality and Radiation in Chernobyl Birds
Source: PLoS One. 2014 Jun 25;9(6):e100296. doi: 10.1371/journal.pone.0100296 (PMC4070951; doi:10.1371/journal.pone.0100296)
Supplement: Table S1 — Bird species and number of individuals with aspermy and with sperm around Chernobyl. (DOC) [file pone.0100296.s001.doc]

**Table S1** Bird species and number of individuals with aspermy and sperm sampled in Chernobyl. Species trapped at least at two different sites are shown in bold.

| Species | Aspermy | Sperm | Totalt |
| --- | --- | --- | --- |
| *Acrocephalus arundinaceus* | 0 | 1 | 1 |
| *Acrocephalus palustris* | 0 | 2 | 2 |
| *Acrocephalus scirpaceus* | 1 | 1 | 2 |
| *Aegithalos caudatus* | 1 | 0 | 1 |
| ***Anthus trivialis*** | **4** | **8** | **12** |
| *Carduelis carduelis* | 0 | 1 | 1 |
| *Carduelis chloris* | 1 | 0 | 1 |
| ***Certhia familiaris*** | **0** | **4** | **4** |
| ***Coccothraustes coccothraustes*** | **2** | **23** | **25** |
| *Delichon urbica* | 0 | 1 | 1 |
| ***Emberiza citrinella*** | **0** | **7** | **7** |
| *Emberiza schoeniclus* | 0 | 1 | 1 |
| ***Erithacus rubecula*** | **5** | **40** | **45** |
| ***Ficedula hypoleuca*** | **0** | **2** | **2** |
| *Ficedula parva* | 0 | 1 | 1 |
| ***Fringilla coelebs*** | **7** | **46** | **53** |
| *Garrulus glandarius* | 1 | 1 | 2 |
| *Hippolais icterina* | 3 | 9 | 12 |
| ***Hirundo rustica*** | **3** | **115** | **118** |
| ***Lanius collurio*** | **10** | **13** | **23** |
| *Lullula arborea* | 0 | 2 | 2 |
| ***Luscinia luscinia*** | **3** | **14** | **17** |
| ***Motacilla alba*** | **0** | **11** | **11** |
| *Muscicapa striata* | 0 | 1 | 1 |
| *Oriolus oriolus* | 0 | 1 | 1 |
| ***Parus caeruleus*** | **2** | **5** | **7** |
| *Parus cristatus* | 4 | 3 | 7 |
| ***Parus major*** | **4** | **35** | **39** |
| *Parus montanus* | 0 | 3 | 3 |
| ***Phoenicurus ochruros*** | **1** | **4** | **5** |
| *Phoenicurus phoenicurus* | 0 | 1 | 1 |
| ***Phylloscopus collybita*** | **2** | **6** | **8** |
| ***Phylloscopus sibilatrix*** | **0** | **19** | **19** |
| ***Phylloscopus trochilus*** | **2** | **5** | **7** |
| *Pyrrhula pyrrhula* | 0 | 1 | 1 |
| *Sturnus vulgaris* | 1 | 0 | 1 |
| ***Sylvia atricapilla*** | **1** | **20** | **21** |
| *Sylvia borin* | 0 | 2 | 2 |
| ***Sylvia communis*** | **6** | **3** | **9** |
| *Sylvia curruca* | 0 | 3 | 3 |
| ***Sylvia nisoria*** | **4** | **6** | **10** |
| ***Troglodytes troglodytes*** | **0** | **3** | **3** |
| ***Turdus merula*** | **4** | **41** | **45** |
| ***Turdus philomelos*** | **2** | **19** | **21** |
| *Turdus pilaris* | 0 | 2 | 2 |
| *Turdus viscivorus* | 2 | 4 | 6 |
| Totalt | 76 | 490 | 566 |
