# Supplementary material for: Aspermy, Sperm Quality and Radiation in Chernobyl Birds
Source: PLoS One. 2014 Jun 25;9(6):e100296. doi: 10.1371/journal.pone.0100296 (PMC4070951; doi:10.1371/journal.pone.0100296)
Supplement: Table S2 — Bird species and number of individuals sampled for sperm around Chernobyl. (DOC) [file pone.0100296.s002.doc]

|  |  |  |  |
| --- | --- | --- | --- |
|  |  |  |  |
|  |  |  |  |
|  |  |  |  |
|  |  |  |  |
|  |  |  |  |
|  |  |  |  |
|  |  |  |  |
|  |  |  |  |
|  |  |  |  |
|  |  |  |  |
|  |  |  |  |
|  |  |  |  |
|  |  |  |  |
|  |  |  |  |
|  |  |  |  |
|  |  |  |  |
|  |  |  |  |
|  |  |  |  |
|  |  |  |  |
|  |  |  |  |
|  |  |  |  |
|  |  |  |  |
|  |  |  |  |
|  |  |  |  |
|  |  |  |  |
|  |  |  |  |
|  |  |  |  |
|  |  |  |  |
|  |  |  |  |
|  |  |  |  |
|  |  |  |  |
|  |  |  |  |
|  |  |  |  |
|  |  |  |  |
|  |  |  |  |
|  |  |  |  |
|  |  |  |  |
|  |  |  |  |
|  |  |  |  |
|  |  |  |  |
|  |  |  |  |
|  |  |  |  |
|  |  |  |  |
|  |  |  |  |
|  |  |  |  |
|  |  |  |  |
|  |  |  |  |

**Table S2** Number of males (N) in species for which we successfully obtained a sperm sample for at least two trapping sites. Mean, minimum and maximum background radiation (μSv/h) for each site and species are also given.

| Species | Bobor | Dytiatki | Fish | Kristatitse | Red Forest | Rudnia | Vesniane | Voronkov | Total N | Mean radiation (min-max) |
| --- | --- | --- | --- | --- | --- | --- | --- | --- | --- | --- |
| *Anthus trivialis* | 0 | 0 | 0 | 0 | 9 | 0 | 1 | 0 | 10 | 34.14 (7.10-85.28) |
| *Certhia familiaris* | 0 | 0 | 0 | 2 | 0 | 0 | 1 | 0 | 3 | 2.05 (0.2-6.11) |
| *Coccothraustes coccothraustes* | 5 | 0 | 0 | 5 | 5 | 0 | 2 | 0 | 17 | 8.54 (0.02-31.31) |
| *Emberiza citrinella* | 2 | 1 | 0 | 1 | 0 | 0 | 1 | 0 | 5 |  |
| *Erithacus rubecula* | 2 | 0 | 4 | 9 | 14 | 0 | 9 | 0 | 38 | 15.16 (0.02-73.48) |
| *Ficedula hypoleuca* | 0 | 0 | 0 | 1 | 1 | 0 | 0 | 0 | 2 |  |
| *Fringilla coelebs* | 1 | 0 | 1 | 8 | 20 | 0 | 5 | 0 | 35 | 15.37 (0.02-52.38) |
| *Hippolais icterina* | 1 | 0 | 1 | 1 | 1 | 0 | 0 | 0 | 4 |  |
| *Hirundo rustica* | 1 | 16 | 0 | 0 | 0 | 17 | 15 | 56 | 105 | 0.41 (0.02-2.90) |
| *Lanius collurio* | 5 | 0 | 1 | 0 | 5 | 1 | 1 | 0 | 13 | 14.58 (0.05-84.00) |
| *Luscinia luscinia* | 5 | 0 | 3 | 0 | 2 | 0 | 0 | 0 | 10 | 5.38 (1.119-12.11) |
| *Motacilla alba* | 2 | 0 | 2 | 0 | 4 | 1 | 0 | 0 | 9 | 15.33 (3.69-21.19) |
| *Parus caeruleus* | 0 | 0 | 1 | 1 | 1 | 0 | 0 | 0 | 3 |  |
| *Parus major* | 3 | 0 | 2 | 8 | 14 | 0 | 4 | 0 | 31 | 6.92 (0.02-29.75) |
| *Phoenicurus ochruros* | 1 | 0 | 1 | 0 | 0 | 0 | 1 | 0 | 3 |  |
| *Phylloscopus collybita* | 1 | 0 | 0 | 1 | 3 | 0 | 0 | 0 | 5 | 0.45 (0.02-.0.88) |
| *Phylloscopus sibilatrix* | 0 | 0 | 0 | 6 | 9 | 0 | 1 | 0 | 16 | 25.35 (0.02-77.28) |
| *Phylloscopus trochilus* | 0 | 0 | 0 | 0 | 2 | 0 | 4 | 0 | 6 |  |
| *Sylvia atricapilla* | 5 | 0 | 0 | 4 | 0 | 0 | 1 | 0 | 10 |  |
| *Sylvia communis* | 0 | 0 | 1 | 0 | 1 | 0 | 0 | 0 | 2 |  |
| *Sylvia nisoria* | 3 | 0 | 0 | 0 | 4 | 0 | 0 | 0 | 7 | 6.38 (0.68-15.16) |
| *Troglodytes troglodytes* | 0 | 0 | 1 | 0 | 1 | 0 | 0 | 0 | 2 |  |
| *Turdus merula* | 6 | 0 | 2 | 7 | 15 | 0 | 5 | 0 | 35 | 19.14 (0.02-86.55) |
| *Turdus philomelos* | 1 | 0 | 1 | 8 | 7 | 0 | 2 | 0 | 19 | 13.61 (0.02-72.01) |
| Mean radiation (min-max) | 0.76 (0.04-4.25) | 0.05 (0.05-0.05) | 3.66 (0.10-7.19) | 0.02  (0.02-0.02) | 27.30 (0.98-137.90) | 0.05 (0.05-0.05) | 6.29 (0.60-9.21) | 0.02  (0.02-0.02) |  |  |
