# Supplementary material for: Aspermy, Sperm Quality and Radiation in Chernobyl Birds
Source: PLoS One. 2014 Jun 25;9(6):e100296. doi: 10.1371/journal.pone.0100296 (PMC4070951; doi:10.1371/journal.pone.0100296)
Supplement: Table S3 — Principal component analysis of 13 sperm parameters recorded with CASA. Rotation method: Varimax with Kaiser Normalization. Loadings above 0.4 are shown in bold. (DOC) [file pone.0100296.s003.doc]

**Table S3** Principal component analysis of 13 sperm parameters recorded by CASA. We used the Varimax rotation method with Kaiser Normalization. Loadings above +0.4 are shown in bold.

|  | PC1 | PC2 | PC3 |
| --- | --- | --- | --- |
| Eigenvalue | 5.15 | 3.15 | 1.57 |
| Percent variance | 39.58 | 24.25 | 12.05 |
| Cumulative percent variance | 39.58 | 63.83 | 75.88 |
| VAP | **0.931** | 0.222 | -0.105 |
| VSL | **0.911** | 0.267 | -0.068 |
| VCL | **0.914** | 0.014 | -0.115 |
| ALH | **0.570** | -0.281 | -0.166 |
| BCF | 0.174 | -0.128 | **0.702** |
| STR | **0.686** | 0.189 | **0.557** |
| LIN | **0.698** | **0.402** | 0.355 |
| MOT | 0.138 | **0.970** | 0.091 |
| PROG | 0.265 | **0.933** | -0.057 |
| RAPID | 0.265 | **0.928** | -0.085 |
| MED | -0.192 | **0.706** | **0.444** |
| SLOW | -0.222 | **0.511** | **0.599** |
| STAT | -0.212 | 0.064 | **0.567** |

|  |  |  |  |  |  |
| --- | --- | --- | --- | --- | --- |
|  |  |  |  |  |  |
|  |  |  |  |  |  |
|  |  |  |  |  |  |
