# Supplementary material for: Aspermy, Sperm Quality and Radiation in Chernobyl Birds
Source: PLoS One. 2014 Jun 25;9(6):e100296. doi: 10.1371/journal.pone.0100296 (PMC4070951; doi:10.1371/journal.pone.0100296)
Supplement: Table S4 — Repeatability ( R ) of the three principal components of sperm behavior. (DOC) [file pone.0100296.s004.doc]

**Table S4** Repeatability (*R*) of the three principal components of sperm behavior.

|  | *R* | SE | *F* | d.f. | *P* |
| --- | --- | --- | --- | --- | --- |
| PC 1 | 0.126 | 0.016 | 3.38 | 23, 375 | < 0.0001 |
| PC 2 | 0.157 | 0.018 | 3.97 | 23, 375 | < 0.0001 |
| PC 3 | 0.039 | 0.010 | 1.67 | 23, 375 | 0.029 |
